# Follicle flushing in monofollicular in vitro fertilization almost doubles the number of transferable embryos

**DOI:** 10.1111/aogs.12054

**Published:** 2012-12-21

**Authors:** Michael Wolff, Yu-Zhen Hua, Alessandro Santi, Erika Ocon, Benedicte Weiss

**Affiliations:** Department of Gynecological Endocrinology and Reproductive Medicine, University Women's HospitalBern, Switzerland

**Keywords:** Embryo, follicle flushing, in vitro fertilization, natural cycle in vitro fertilization, oocyte

## Abstract

Follicle flushing has been proved to be ineffective in polyfollicular in vitro fertilization. To analyze the effect of flushing in monofollicular in vitro fertilization we aspirated and then flushed the follicles in 164 cycles. Total oocyte yield/aspiration was 44.5% in the aspirate, 20.7% in the 1^st^ flush, 10.4% in the 2^nd^ flush and 4.3% in the 3^rd^ flush. By flushing, the total oocyte yield increased (p < 0.01) by 80.9%, from 44.5 to 80.5%. The total transfer rate increased (p < 0.01) by 91.0%, from 20.1 to 38.4%. The results indicate that the oocyte yield and the number of transferable embryos can be increased significantly by flushing.

## Introduction

Follicle flushing has been shown to be ineffective in increasing the number of aspirated oocytes. The last Cochrane analysis concluded that “there is no evidence that follicular aspiration and flushing is associated with … an increase in oocyte yield”. Follicular flushing even seemed to be disadvantageous, as “the operative time is significantly longer and more opiate analgesia is required for pain relief during oocyte retrieval” 1. This statement was based on different studies in which follicle aspiration was performed in polyfollicular in vitro fertilization (IVF) (normal responders) and oligofollicular IVF (low responders) following controlled ovarian hyperstimulation. However, many IVF centers have introduced monofollicular IVF, as this technique promises to have some advantages in certain groups of patients 2. Therefore it remains to be evaluated if this statement also applies to monofollicular IVF.

## Material and methods

In all, 164 aspirations were performed in monofollicular IVF-cycles in 2011, following written consent. Monofollicular IVF was defined as natural cycle IVF (n = 68) (including 5000 units human chorionic gonadotropin, hCG), IVF with low doses of clomiphene citrate (n = 77) (including clomiphene citrate 25 mg/day from day 6 until induction of ovulation plus 5000 units hCG) and IVF with low doses of human menopausal gonadotropin (n = 15) (including 75 units human menopausal gonadotropin from day 6 until induction of ovulation plus 5000 units hCG). hCG was given 36 h before aspiration. Where luteinizing hormone was already increased at the time of follicle monitoring, follicle aspiration was performed without hCG. Only those cycles with one follicle were analyzed.

Follicles were aspirated without anesthesia and analgesia, using gauge 19 single lumen needles (250 mm Hg) as described elsewhere 3. After initial aspiration, follicles were flushed and aspirated three times each with 2 mL flushing medium with heparin (SynVitro® Flush, Origio, Berlin, Germany). Fertilization was achieved by standard intracytoplasmic sperm injection. The oocyte yield, the proportion of mature oocytes, the fertilization rate (pronuclear stage) and the transfer rate were analyzed for each aspiration step. Embryos were transferred two to three days after aspiration. Fisher's exact test was used to analyze the difference in oocyte numbers and transfer rates.

## Results

Average patient age was 37.0 years (SD ± 3.8, range 28–45) and basal levels of follicle stimulating hormone were between 2.0 and 47.4 (average 9.1 ± 8.2). Overall cancellation rate due to premature ovulation was 20/115 (17.4%). Follicle size at the time of aspiration was 19.0 ± 2.1 mm. Follicle aspiration took around 30 sec. Flushing extended the aspiration time by less than 1 min.

Total oocyte yield/aspiration was 44.5% in the aspirate, 20.7% in the 1^st^ flush, 10.4% in the 2^nd^ flush and 4.3% in the 3^rd^ flush (Table 1). In each aspiration step, the oocyte yield was reduced by around 50%. By flushing, the total oocyte yield increased significantly (p < 0.01) by 80.9%, from 44.5 to 80.5%.

**Table 1:** Total oocyte yield, proportion of mature oocytes and fertilization rate in follicular aspirates and in different flushings in monofollicular in vitro fertilization (IVF)

	Monofollicular IVF				
Aspirations, n			164	
Mean age, years ± SD			37.0 ± 3.8 (28–45)	
Mean follicular diameter, mm ± SD			19.0 ± 2.1	
Total oocytes/aspirations, n			132/164	
Total oocytes/aspirations, %			80.5	

	Aspirate	1^st^ flush	2^nd^ flush	3^rd^ flush
Oocytes/group/aspirations, n	73/164	34/1645	17/164	7/164
Oocytes/group/aspirations, %	44.5	20.7	10.4	4.3
MII oocytes/aspirated oocytes, n	67/73	31/34	16/17	7/7
MII oocytes/aspirated oocytes, %	91.8	91.2	94.1	100
Fertilized oocytes/MII oocytes, n	33/67	16/31	10/16	4/7
Fertilized oocytes/MII oocytes, %	49.3	51.6	62.5	57.1
Fertilized oocytes/aspirated oocytes, n	33/73	16/34	10/17	4/7
Fertilized oocytes/aspirated oocytes, %	45.2	47.1	58.8	57.1
Transfer rate/group/aspiration, n	33/164	16/164	10/164	4/164
Transfer rate/group/aspiration, %	20.1	9.8	6.1	2.4
Total transfer rate/aspiration, n	63/164			
Total transfer rate/aspiration, %	38.4			

The proportions of metaphase II oocytes/aspirated oocytes were similar in all four groups (91.8, 91.2, 94.1 and 100%). The proportion of fertilized (pronuclear) oocytes/ metaphase II oocytes were also similar in all four groups (49.3, 51.6, 62.5 and 57.1%) as well as the proportion of fertilized (pronuclear)/aspirated oocytes (45.2, 47.1, 58.8 and 57.1%).

Transfer rates in each group/total aspirations were 20.1, 9.8, 6.1 and 2.4%. By flushing, the total transfer rate increased significantly (p < 0.01) by 91.0%, from 20.1 to 38.4%. As the transfer rate dropped to 2.4% in the last flushing step, we defined a maximum of three flushing steps to be useful. Clinically relevant bleeding, injury to pelvic organs or peritoneal infection was never observed.

## Discussion

The history of IVF shows a progression from the original monofollicular IVF to polyfollicular IVF, sometimes with a very high number of follicles. However, the number of follicles has once again declined in many centers, partly because of a reduction in the stimulation dose and partly because of the increasing average age of the patients. Specialists in reproductive medicine are therefore increasingly being confronted with an oligofollicular reaction. Irrespective of this, monofollicular natural cycle IVF is being increasingly propagated as a treatment method. With the increase in oligo- and monofollicular IVF techniques, a re-evaluation of the aspiration techniques is necessary.

In this study, we could show that follicle flushing is beneficial, at least in monofollicular IVF. Further studies will show whether our observations can be extended to oligofollicular IVF. When interpreting our results, it should be considered that there is a certain possibility that the oocytes remain in the aspiration needle during the aspiration and are therefore washed back into the follicle during flushing. The needle and aspiration tubing had a capacity of 0.7 mL in our system. This is equivalent to about 15% of the mean follicle volume of approximately 4 mL, which is aspirated from a follicle size of 20 mm. The probability of the oocytes remaining in the needle and tubing is therefore about 15%. The oocyte retrieval rate from the aspiration may thus be calculated to be 6.7% higher than the 44.5% stated in our study when the needle is removed and completely evacuated after the aspiration. Nevertheless, even with this calculation, washing the follicle is clearly superior to simple aspiration.

In the Cochrane analysis mentioned above 1, it was shown that flushing would lead to a longer duration of anesthesia, which can be disadvantageous for the oocytes as well as for the patient. As aspiration of a single follicle is unproblematic without anesthesia and possible without analgesia 3, this argument does not apply to monofollicular IVF. The three flushings prolonged the aspiration time by less than 1 min in our study. The flushing itself was not painful. Our aspiration technique was stated by 85% of surveyed women to be as or less painful than having a blood sample taken 3.

The effect of follicle flushing has also been described in a French publication 4. Mendez Lozano et al. performed an aspiration without flushing in 79 women and with triple flushing in 47 women with a total of 10 mL flushing medium. The percentage of patients with a good embryo was 28.8% in the group without flushing and 37.8% in the group with flushing; however, the difference was not significant. The effect of the individual flushes was not analyzed. These studies basically showed similar results to ours. However, no significance could be assigned and additional detailed data from the individual flushes were not ascertained.

In conclusion, three flushings almost doubled not only the number of aspirated oocytes but also the transfer rate in monofollicular IVF. These results indicate, first, that the oocyte yield can be increased by flushing and, secondly, that the oocytes, collected by flushing, are as mature and fertilizable as those aspirated without flushing.

## Funding

No special funding.

## Abbreviations

hCG: human chorionic gonadotropin
IVF: in vitro fertilization
